# Sodium-Glucose Cotransporter 2 Inhibitors Potentially Prevent Atrial Fibrillation by Ameliorating Ion Handling and Mitochondrial Dysfunction

**DOI:** 10.3389/fphys.2020.00912

**Published:** 2020-08-04

**Authors:** Xiaodong Peng, Linling Li, Mengxia Zhang, Qianqian Zhao, Kui Wu, Rong Bai, Yanfei Ruan, Nian Liu

**Affiliations:** Department of Cardiology, Beijing Anzhen Hospital, Capital Medical University, Beijing, China

**Keywords:** sodium-glucose cotransporter 2 inhibitors, atrial fibrillation, calcium handling, sodium-hydrogen exchanger isoform 1, mitochondria

## Abstract

Sodium-glucose cotransporter 2 inhibitors (SGLT2i) are a novel class of glucose-lowering agents that significantly improve the prognosis of patients with type 2 diabetes (T2D) and heart failure. SGLT2i has recently been implicated in the treatment of atrial fibrillation (AF) with clinical data demonstrating that these agents decrease the incidence of AF events in patients with T2D. Fundamental findings have suggested that SGLT2i may alleviate atrial electrical and structural remodeling. The underlying mechanisms of SGLT2i are likely associated with balancing the sodium and calcium handling disorders and mitigating the mitochondrial dysfunction in atrial myocytes. This review illustrates the advances in understanding the underlying mechanisms of SGLT2i as an evolving treatment modality for AF.

## Introduction

Atrial fibrillation (AF) is a prominent public health and economic issue globally. It is estimated that about 33.5 million people worldwide suffered from AF in 2010, and the number of affected patients is predicted to reach 12.1 and 14–17 million by the year 2030 in the United States and Europe, respectively, exerting tremendous pressure on the global economy (Colilla et al., 2013; Chugh et al., 2014; Zoni-Berisso et al., 2014). In clinical practice, AF is often comorbid with heart failure (HF) and type 2 diabetes (T2D), accelerating the progression of these diseases. Moreover, patients with AF have an increased (fivefold) risk of suffering from stroke, resulting in severe disability or death (Pistoia et al., 2016).

Some progress has been made in our understanding of the pathophysiology of AF. The ectopic beats from the pulmonary veins are called “triggers,” considered to be signals for the initiation of AF (Haïssaguerre et al., 1998; Santangeli and Marchlinski, 2017). Therefore, pulmonary vein isolation by insulating pulmonary veins from the atrium becomes the cornerstone of catheter ablation for the prevention of AF. The “substrate” has been well-recognized for supporting and maintaining AF, which is caused by electrical and structural remodeling in the atrium (Allessie et al., 2002). In addition to the deposition of elastic fibers and collagen in the atrium, the arrhythmogenesis of atrial myocytes has gained increasing attention in the field. In atrial myocytes, the energetic deficits and oxidative stress caused by mitochondrial dysfunction and alterations in Na^+^ and Ca^2+^ handling are critical pathophysiological hallmarks (Poulet et al., 2015; Dobrev and Wehrens, 2017; Karam et al., 2017). Amelioration of these deficits is considered a promising therapeutic strategy for the treatment of AF.

**FIGURE 1:**
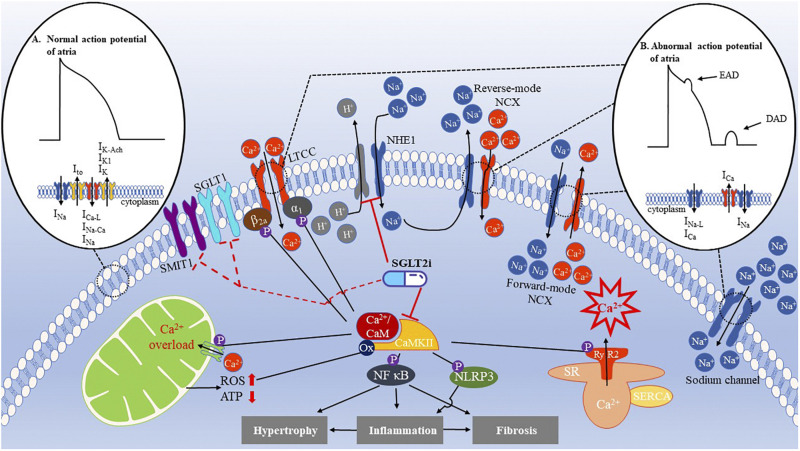
Ion disorder and mitochondrial dysfunction in AF and cardioprotective effects of SGLT2i. **(A)** The normal action potential (AP) of atria is initiated by the rapid influx of Na^+^ current (I_Na_) to form an upstroke AP. Subsequently, transient outward current (I_to_) achieves rapid repolarization, followed by inward L-type Ca^2+^ currents (I_Ca–L_), I_Na_, and Na^+^/Ca^2+^ exchange current (I_Na–Ca_) as well as outward currents, such as inward rectifying potassium (I_K1_), delayed rectifier potassium (IK), and acetylcholine-sensitive potassium (I_K–Ach_) currents, which maintain the AP plateau. Afterward, I_K1_, I_K_, and I_K–Ach_ completely achieve repolarization. This period is called the action potential duration (APD). **(B)** Abnormal AP is caused by irregular ion handling. For instance, increased late Na^+^ current (I_Na–L_) and Ca^2+^ current (I_Ca_) during the AP plateau or three phases can shorten the APD and generate early after-depolarizations (EAD). Moreover, excessive Ca^2+^ release from the SR activates the forward-mode NCX and increases the inward currents leading to delayed after-depolarizations (DADs). In patients with AF, the NHE1 is upregulated, enhancing the influx of Na^+^. As a result, intracellular Ca^2+^ levels increase, effectively impacting the inflammatory, fibrosis, and hypertrophic pathways that contribute to atrial remodeling. CaMKII is an important regulator of Ca^2+^ handling, which phosphorylates the ryanodine receptor 2 (RyR2) to exacerbate the Ca^2+^ leak from the SR, causing a Ca^2+^ overload. In addition, CaMKII also phosphorylates the L-type Ca^2+^ channel (LTCC) Ca_V_1.2 β_2a_ subunits and Ca_V_1.3 α_1_ subunit to increase Ca^2+^ entry, activating the NF-κB and NLRP3 inflammasomes to initiate hypertrophy, inflammation, and fibrosis of atria. Mitochondrial Ca^2+^ uniporter (MCU) is phosphorylated by CaMKII, which causes a Ca^2+^ overload in the mitochondria, producing more ROS, which, in turn, oxidizes CaMKII to further activate CaMKII. These effects can be eliminated by SGLT2i. SGLT1 and sodium-myoinositol cotransporter 1 (SMIT1) are potential targets of SGLT2i.

Sodium-glucose cotransporter 2 inhibitors (SGLT2i) are novel glucose-lowering agents used in the treatment of T2D. SGLT2 is a low-affinity, high-capacity glucose transporter located in the proximal tubule in the kidneys and is responsible for glucose reabsorption into the body from urine (Scheen, 2014). The (Empagliflozin) Cardiovascular Outcome Event Trial in Type 2 Diabetes Mellitus Patients (EMPA-REG OUTCOME), a breakthrough in the treatment of T2D, revealed that empagliflozin reduces the risk of major adverse cardiovascular events by 38% relative to placebo (Zinman et al., 2015). In the subgroup analysis of the EMPA-REG OUTCOME trial, patients with AF especially benefited from the use of empagliflozin compared with patients without AF (Böhm et al., 2020). Moreover, the Multicenter Trial to Evaluate the Effect of Dapagliflozin on the Incidence of Cardiovascular Events (DECLARE-TIMI 58) has shown that dapagliflozin decreases the incidence of reported episodes of AF events in high-risk patients with T2D (Zelniker et al., 2019). A cohort study that enrolled more than 150,000 cases demonstrated that patients with T2D treated with SGLT2i were less likely to develop AF (Chen et al., 2020). A recent meta-analysis incorporating 10 studies published between 2014 and 2019 revealed that AF was significantly reduced after the management of SGLT2i (Cheung et al., 2020). Accumulating evidence suggests that the cardioprotective effects of SGLT2i are independent of their glucose-lowering properties (Juni et al., 2019; Li et al., 2019). Currently, SGLT2i is thought to balance Na^+^ and Ca^2+^ homeostasis and rescue mitochondrial function. In this review, we discuss the potential therapeutic mechanisms of SGLT2i as novel targets for the treatment of AF (Figure 1).

## Na^+^ and Ca^2+^ Handling in Healthy and Fibrillating Atrium: Therapeutic Target of Sodium-Glucose Cotransporter 2 Inhibitors

### Normal Na^+^ and Ca^2+^ Handling in Cardiac Myocytes

The delicate balance of Na^+^ and Ca^2+^ fluxes between the intracellular and extracellular spaces in cardiac myocytes is pivotal to ensure normal contractile function and cardiac rhythm. During the plateau phase of the cardiac action potential, a small amount of Ca^2+^ enters the cardiac myocytes through L-type Ca^2+^ channels, causing a large Ca^2+^ release into the cytosol via the RyR2 channel. This channel is located in the membrane of the sarcoplasmic reticulum (SR), which is called Ca^2+^-induced Ca^2+^ release (CICR) (Ibrahim et al., 2012). The cytosolic Ca^2+^ increases to 1 μM from 150 nM in baseline and activates the contractile apparatus (Bers, 2002). This process is also named cardiac excitation-contraction (E-C) coupling. Next, the elevated cytosolic Ca^2+^ needs to be promptly resumed to 150 nM during the diastolic period to maintain the normal relaxation property of cardiac myocytes. The majority of Ca^2+^ is taken up again into the SR by sarcoplasmic/endoplasmic reticulum Ca^2+^ ATPase isoform 2a (SERCA2a) (Bers, 2008). The remaining Ca^2+^ is mainly extruded into the extracellular fluid via a forward-mode Na^+^/Ca^2+^ exchanger (NCX).

Na^+^ hemostasis in cardiac myocytes is regulated by the Na^+^ channels and transporters that couple the Na^+^ influx to either co- or countertransport of other ions, such as NCX, Na^+^/H^+^ exchange (NHE), and Na^+^/K^+^-ATPase (NKA). Na^+^ channels and NCX are the dominant Na^+^ influx and efflux pathways in the atrium, respectively. There is a tight coupling between Na^+^ and Ca^2+^ homeostasis via the NCX in the microdomain beneath the T-tube. The NCX may operate in both forward and reverse directions, depending on the electrochemical gradients and membrane potential. Elevated intracellular Ca^2+^ levels activate the forward mode of NCX, extruding the Ca^2+^ in exchange for Na^+^ with a stoichiometry of 1:3 that generates a net inward current. The transient inward current results in delayed after-depolarizations (DADs) during the diastolic period and triggers activity once it reaches the threshold of the Na^+^ channel (Voigt et al., 2012). Na^+^ elevation in the cytosol may limit Ca^2+^ extrusion via the forward mode of NCX and could even facilitate Ca^2+^ influx through the reverse mode (Baartscheer et al., 2003). Both scenarios suggest that elevated cytosolic Na^+^ enhances intracellular Ca^2+^ overload via the NCX pathway, subsequently resulting in cardiac dysfunction and arrhythmogenesis.

### Abnormal Na^+^ and Ca^2+^ Handling During Atrial Fibrillation

A growing body of evidence has shown that the development and maintenance of AF are associated with dysregulation of the Ca^2+^ and Na^+^ handling in atrial myocytes. Ca^2+^ handling disorder may promote triggered activity, conduction abnormalities, and atrial remodeling (Jalife and Kaur, 2015). The latter two points may facilitate reentry and form the substrate for the maintenance of AF. In patients with and animal models of AF, the Ca^2+^/calmodulin-dependent protein kinase type-II (CaMKII) complex is activated and phosphorylates several Ca^2+^-handling proteins, such as RyR2 and phospholamban (PLN) in atrial myocytes. This enhances the Ca^2+^ leak from the SR, resulting in the triggered activity that serves as the initiation of AF (Li et al., 2012). Elevated cytosolic Ca^2+^ levels may promote profibrotic and inflammatory pathways that contribute to atrial remodeling, accelerating the formation of the substrate for AF. For instance, Ca^2+^ mobilization is crucial for the assembly and activation of an inflammatory signaling complex called the NLRP3 inflammasome (Murakami et al., 2012). NLRP3 exacerbates the Ca^2+^ leak from the SR, leading to ectopic firing in the atrium and enhances pyroptosis mediated by caspase-1, accelerating the formation of AF-maintaining substrates. In addition, intracellular Ca^2+^ leaks disturb mitochondrial function, leading to an increase in reactive oxygen species (ROS) production and impairment of energetic metabolism, resulting in the oxidation of the calcium-handling proteins, such as RyR2 and exacerbation of the Ca^2+^ leak, subsequently promoting the initiation and maintenance of AF.

Na^+^ overload underlies the development of AF. The late Na^+^ current (I_Na–L_) is enhanced in AF, driving NCX in the reverse mode and increasing the cytosolic Ca^2+^ levels (Song et al., 2008). Normalizing Na^+^ overload by selectively suppressing I_Na–L_, such as ranolazine, is a promising therapeutic strategy for AF. Notably, activation of NHE promotes cardiac hypertrophy and HF via the Na^+^ overload-induced Ca^2+^ overload pathway (Karmazyn et al., 2008). In the atrial tachycardia-induced canine model, NHE is upregulated and the NHE1 inhibitor prevents effective refractory period abbreviations, delaying the development of atrial contractile dysfunction (Jayachandran et al., 2000). The underlying mechanisms are likely associated with the alleviation of the harmful consequences of increased cytosolic Na^+^ levels.

### The Protective Effect of Sodium-Glucose Cotransporter 2 Inhibitors on Disorganized Ca^2+^/Na^+^ Balance

SGLT2i inhibits SGLT2 in the proximal tubules to prevent the reabsorption of glucose and Na^+^, facilitating their excretion in urine. SGLT2i may decrease the cytosolic Na^+^ levels at a tissue and cellular level due to its pharmacological action. Karg et al. (2018) reported that SGLT2 inhibition with dapagliflozin resulted in a significant decrease in the Na^+^ content of the skin. Moreover, I_Na–L_ was remarkably reduced in empagliflozin-treated T2D rats (Lee et al., 2019). In isolated ventricular myocytes, Baartscheer et al. (2017) observed that empagliflozin significantly decreased intracellular Na^+^ and Ca^2+^ levels within 10 min. This effect was not influenced by extracellular glucose levels. In line with the lack of SGLT2 in cardiac myocytes, it is speculated that SGLT2 is not responsible for the action of empagliflozin. Intriguingly, the authors found that pretreatment with cariporide, an NHE1 inhibitor, abolished the effects of empagliflozin. Thus, it is proposed that empagliflozin is a novel NHE1 inhibitor. Subsequently, Uthman et al. (2018a) confirmed that empagliflozin, dapagliflozin, and canagliflozin directly inhibit the cardiac NHE flux and reduce cytosolic Na^+^ levels. Molecular docking studies demonstrated that all three SGLT2i efficiently bind to the Na^+^-binding pocket of NHE with high affinities (Uthman et al., 2018a). The authors postulated that SGLT2i reduces cytosolic Na^+^ levels by blocking NHE, decreasing intracellular Ca^2+^ concentration, and ultimately optimizing cardiac function. In the aging and AF atria, NHE activity is upregulated, which may produce Na^+^ overload and contribute to the development of AF (hui et al., 2008). It is reasonable that SGLT2i can prevent AF by ameliorating Na^+^ overload induced by the upregulated NHE activity in the atrium despite the lack of direct evidence. Ye et al. (2018) also showed that dapagliflozin could increase phosphorylated adenosine monophosphate kinase (AMPK) to reduce the mRNA expression of NHE1 in cardiac fibroblasts. Compelling evidence shows that NHE is a novel target for SGLT2i. Acute inhibition of NHE by SGLT2i may diminish the incidence of the triggered activity induced by Na^+^ overload, which has generally been recognized as the primary mechanism for AF. Long-term inhibition of NHE by SGLT2i may reverse the atrial remodeling and alleviate the substrate for AF maintenance through the alleviation of the abnormal downstream NHE signaling. However, some have pointed out that atrial remodeling could not be prevented through NHE1 inhibition (Blaauw et al., 2004). Further studies are warranted to directly explore the effect of SGLT2i on NHE in the fibrillating atria.

There are two additional members of the SGLT family expressed in the heart. One of them is SGLT1, which is upregulated in some diseases, such as T2D (Velmurugan et al., 2019). SGLT1 contributes to the Na^+^ influx in cardiac myocytes, and this action becomes dominant under pathological conditions. Sawa et al. (2020) report that pretreatment with KGA-2727, a selective SGLT1 inhibitor, protects against myocardial infarction–induced ventricular remodeling and HF in mice. SGLT inhibitors can be more or less selective for SGLT2 vs. SGLT1. The half-maximal inhibitory concentration IC50 of empagliflozin for SGLT1 is 8.3 μmol/L. Thus, SGLT2i may mitigate Na^+^ overload via SGLT1. Sodium-myoinositol cotransporter 1 (SMIT1) is another example that barely regulates glucose uptake under physiological conditions. However, it activates NADPH oxidase 2 (NOX2) and causes Na^+^ overload when it is overexpressed (Van Steenbergen et al., 2017). It is proposed that SGLT2i may benefit cardiac function by targeting SMIT1.

Several studies have shown that acute administration of SGLT2i promptly improves cardiac function in patients with HF. Lehrke et al. (2019) reports that treatment with empagliflozin leads to a rapid and sustained improvement of diastolic function in patients with T2D, independent of hemodynamic changes. In the ventricular strips isolated from patients with HF, Pabel et al. (2018) observed that empagliflozin significantly reduced diastolic tension, whereas systolic force was not changed. Furthermore, in anesthetized HF with preserved ejection fraction (HFpEF) rats, intravenous injection of empagliflozin significantly improved diastolic function, whereas systolic contractility was unaffected. Thus, the direct action of SGLT2i on cardiac myocytes targeting Ca^2+^ handling is speculated. In streptozotocin-induced diabetic rats, Lee et al. (2019) found that chronic administration of empagliflozin for 4 weeks significantly reversed the Ca^2+^ dysregulation in cardiac myocytes, including attenuation of the SR Ca^2+^ leak, an increase in Ca^2+^ transient amplitude, and restoration of SR Ca^2+^ content. Next, they revealed that decreased phosphorylated RyR2 at serine 2808 (RyR2-pS2808) and increased protein expression of SERCA2a were responsible for the action of empagliflozin. In the ventricular myocytes isolated from patients with HF, Mustroph et al. (2018) observed that exposure to empagliflozin for 24 h remarkably downregulated the activity of CaMKII. Consequently, the detrimental effects of CaMKII overactivity on Ca^2+^ handling in the failing ventricular myocytes were reversed. It is notable that acute (30 min) empagliflozin exposure did not alter CaMKII activity and did not improve Ca^2+^ handling disorder in failing ventricular myocytes, whereas the elevated cytosolic Na^+^ levels were attenuated. Mustroph et al. (2018) suggest that the direct inhibitory effects of empagliflozin on CaMKII or RyR2 were unlikely, and it may be initiated by the attenuation of Na^+^ overload.

Atrial fibrosis plays an essential role in the pathophysiology of AF, especially for the maintenance of AF (Nattel, 2017). SGLT2i ameliorates adverse cardiac fibrosis and remodeling in patients and animal models (Lee et al., 2017; Uthman et al., 2018b). Recently, Shao et al. (2019) report that empagliflozin significantly reduced the incidence of AF induced by the burst pacing in a rat model of T2D. The underlying mechanism is related to the amelioration of atrial structural and electrical remodeling by empagliflozin. Several studies have shown that the CaMKII cascade on Ca^2+^ regulation may play an essential role in the atrial remodeling of AF (Mustroph et al., 2017). Byrne et al. (2020) showed that empagliflozin attenuated the NLRP3 level and then mitigated the inflammatory response in mice with HF, reducing cardiac fibrosis and remodeling. Sato et al. (2018) observed that treatment with dapagliflozin might improve systemic metabolic parameters and decrease the epicardial adipose tissue volume in T2D patients, which is highly associated with the increasing incidence and severity of AF. Another obesity study in a rabbit model demonstrated that obesity caused atrial remodeling with prolonged refractoriness, upregulated calcium-handling proteins, and advanced fibrosis. Treatment with SGLT2i prevented this remodeling and led to decreased atrial arrhythmogenesis (Cheng et al., 2020).

## Mitochondria and Atrial Fibrillation: Sodium-Glucose Cotransporter 2 Inhibitors Play a Role in Dissolving the Contact

### Mitochondrial Function and Ca^2+^ Cycling

In mitochondria, acetyl-CoA derived from glucose initiates the tricarboxylic acid cycle (TAC), which produces the ATP used for cellular activities. At the same time, ROS that causes an imbalance in ion handling and deterioration of cellular function is also generated through oxidative phosphorylation. Ca^2+^ is considered a vital regulator of mitochondrial metabolism. Mitochondrial Ca^2+^ uniporter (MCU) anchored on the inner mitochondrial membrane mainly takes charge of Ca^2+^ uptake from the cytoplasm during diastole. The mitochondrial membrane potential generated by proton-ATPase drives Ca^2+^ across the inner membrane via MCU. The free energy of ATP hydrolysis (ΔG_ATP_) supports the proton pump to maintain the electrochemical proton gradients and transport of Ca^2+^, which can be disordered when ΔG_ATP_ is decreased due to mitochondrial dysfunction (Rizzuto et al., 2012; Consolini et al., 2017). Hyperactivation of NHE1 is associated with mitochondrial Ca^2+^ overload in the rat model that were subjected to coronary artery ligation during the post-infarction remodeling, which further facilitates the opening of the mitochondrial permeability transition pore (MPTP), increasing mitochondrial fragility (Javadov et al., 2005). In the mitochondria, Ca^2+^ acts as a second messenger to participate in the TAC. On the one hand, Ca^2+^ is necessary for the activation of ATP synthase and enzymes, such as pyruvate dehydrogenase, isocitrate dehydrogenase, and α-ketoglutarate dehydrogenase (O-Uchi et al., 2014). Thus, Ca^2+^ disorders in mitochondria attenuate the electron transport throughout the respiratory chain reducing the ATP production. On the other hand, Ca^2+^-dependent NADH can further generate NADPH, which eliminates accumulating ROS and maintains the balance between oxidation and antioxidation by sustaining a reduced form of glutathione. Meanwhile, Ca^2+^ also activates catalase and glutathione reductase directly to decrease hydrogen peroxide and enhance superoxide dismutase (SOD) (Zhang et al., 2016). Ca^2+^ efflux is primarily mediated by the NCX that is located on the mitochondrial membrane (mitoNCX).

### Mitochondrial Dysfunction and Atrial Fibrillation

AF is tightly connected with the ROS accumulation and increased oxidative stress caused by metabolic dysfunction of mitochondria. Mitochondrial dysfunction participates in AF via the Ca^2+^ signaling-dependent pathway in atrial myocytes (Opacic et al., 2016). Ca^2+^ overload accelerates ROS production by enhancing the electron flow into the respiratory chain and inhibiting complexes I and III on the chain (Brookes et al., 2004). Subsequently, increased ROS oxidizes Ca^2+^ handling proteins, such as RyR2 and CaMKII, inducing the overactivation of these proteins, disrupting Ca^2+^ homeostasis (Mustroph et al., 2017). The latter further facilitates ROS production in mitochondria, resulting in a vicious circle.

The dynamic mitochondrial abnormality is another trait related to AF. Fission and fusion are the two sides of mitochondrial dynamics. During mitochondrial fusion, the outer membranes are fused through the mediation of mitofusins 1/2 (Mfn1/2), which are dependent on GTPases. Second, optic atrophy 1 (OPA1) promotes the fusion of inner membranes. Thus, the exchange of material becomes easier, and mitochondria can continue to sustain their function, including metabolism and ATP production. Mitochondrial membranes are linked to the SR via Mfn2 in AF and when under oxidative stress, forming a construction called mitochondria-associated membrane (MAM), where Ca^2+^ microdomains are created. Ca^2+^ signaling is well-regulated by Mfn2 at the MAM in response to local changes in cytoplasmic Ca^2+^ induced by transient stress. However, mitochondrial fragmentation and cell death are initiated if the rapid stimulation and oxidative stress persist, which may be a target for AF prevention (Redpath et al., 2013).

For fission, dynamin-related protein 1 (Drp1) is recruited to the surface of mitochondria to initiate this process (Meyer et al., 2017). If mitochondrial fusion is prevented, mass transfer between two mitochondria could be ineffective, leading to a metabolic disorder that results in energetic deficits (Chen et al., 2011). Reduced mitochondrial fusion or increased fission would cause mitochondrial dysfunction related to AF. Mitochondrial dynamics are regulated by a subfamily of the Ras GTPases (Miro proteins), which are Ca^2+^-dependent (Reis et al., 2009). The movement of mitochondria can be prevented by the Miro proteins due to Ca^2+^ overload, which further exacerbates the effects of metabolic and dynamic disorders. Other studies have also shown that mitochondrial dysfunction underlies atrial remodeling in experimental and clinical AF. Atrial biopsies from patients with AF display aberrant ATP levels, mitochondrial stress–related chaperone (heat shock protein 10 and 60) upregulation and mitochondrial network fragmentation. Partial blocking or downregulation of MCU may attenuate atrial remodeling by preventing increased mitochondrial Ca^2+^ influx (Wiersma et al., 2019).

### Sodium-Glucose Cotransporter 2 Inhibitors Restore the Metabolic and Dynamic Functions of Mitochondria During Atrial Fibrillation

Improvement of mitochondrial metabolism has been recognized as a therapeutic mechanism of SGLT2i. In a high-fat diet mouse model, canagliflozin protected against obesity-related metabolic disorders by improving mitochondrial function and fatty acid oxidation in adipose tissue (Wei et al., 2020). Sa-Nguanmoo et al. (2017) demonstrated that dapagliflozin remarkably decreased mitochondrial ROS production and mitochondrial membrane depolarization in the brain of obese rats. In ventricular myocytes isolated from overweight insulin-resistant rats, Durak et al. (2018) found that dapagliflozin suppressed oxidative stress in mitochondria reversing the prolonged ventricular-repolarization. SGLT2i also alleviates mitochondrial fusion and fission abnormalities. Increased phosphorylation of Drp1 at S616 and decreased phosphorylation at S637 are strong indicators of overactive mitochondrial fission resulting from myocardial injury (Shen et al., 2018). Empagliflozin increases the AMP/ATP ratio and subsequently activates the AMPK. The AMPK reduces Drp1 phosphorylation at S616 while augmenting the S637 phosphorylation, which suppresses aberrant mitochondrial fission. This enables the resumption of cellular metabolism, improving the diabetic myocardial microvascular injury in mice (Zhou et al., 2018). Empagliflozin also ameliorates the respiratory activities of complex I + II by increasing the uncoupling of the inner membrane, attenuating ROS production, and mitochondrial Ca^2+^ overload following acute ischemic reperfusion injury (Jespersen et al., 2017). Mfn2 and OPA1 protein expression are decreased in mice that are fed high-fat diets, causing tubular cell dilation and detachment. After treating cells with ipragliflozin for 16 weeks, these alterations were reversed (Takagi et al., 2018).

More recently, Shao et al. (2019) provided direct evidence that SGLT2i can restore mitochondrial function, ameliorate electrical and structural remodeling of the atria, and prevent AF in a T2D rat model. The rat model showed an enlarged left atrial diameter and promoted AF inducibility. These detrimental effects were prevented by treatment with empagliflozin for 8 weeks. In the atrial tissue, mitochondrial respiratory function, mitochondrial membrane potential, and energy generation were damaged. Empagliflozin treatment mitigated the mitochondrial biogenesis by upregulating the expression of peroxisome proliferator-activated receptor-c coactivator 1α (PGC-1α), nuclear respiratory factor-1 (NRF-1), and mitochondrial transcription factor A (TFAM). This effectively improved the mitochondrial function. These three proteins are essential parts of mitochondrial DNA (mtDNA) transcription, which regulates ATP synthesis. Reduced ATP contributes to atrial remodeling during AF (Dong et al., 2016). Shao et al. (2019) proposed that the improvement of mitochondrial function and biogenesis in T2D by SGLT2i is responsible for the prevention of T2D-related AF.

To date, the underlying mechanisms of improved mitochondrial function by SGLT2i have not been completely investigated. NHE1 is expressed in the membrane of cardiac mitochondria. Downregulation of NHE1 inhibits Ca^2+^-induced MPTP opening and reduces the release of ROS in ventricular myocytes in patients with HF. As mentioned above, mitochondrial function is tightly influenced by Na^+^ and Ca^2+^ levels in the mitochondria. SGLT2i are novel NHE blockers and are likely involved in the improvement of mitochondrial function in AF by modulating the mitochondrial Na^+^ and Ca^2+^ flux.

## Conclusion

SGLT2i are a new class of diabetic medications used for the treatment of T2D. In the last few years, compelling clinical evidence showed that SGLT2i reduces the incidence of AF in patients with T2D and HF. The potential role of SGLT2i in the treatment of AF is emerging. Based on the current knowledge of AF pathophysiology and the pharmacological actions of SGLT2i, it is speculated that SGLT2i potentially prevents AF by ameliorating ion handling and mitochondrial dysfunction. Understanding the therapeutic mechanisms of SGLT2i in AF has gained considerable interest and has developed rapidly. It is expected that a collaborative effort between clinical and basic science researchers will continue to enable the development of a promising treatment for AF.

## Author Contributions

XP wrote the manuscript. LL, MZ, QZ, KW, RB, and YR took part in preparing the manuscript. NL prepared and reviewed the manuscript before publication. All authors confirmed that they have read and approved the manuscript and they have met the criteria for authorship.

## Conflict of Interest

The authors declare that the research was conducted in the absence of any commercial or financial relationships that could be construed as a potential conflict of interest.
